# The Missing Link

**Published:** 1879-11

**Authors:** 


### Editorial.

### THE MISSING LINK.

An orator, of popular reputation, recently declared that a fact would fit every other fact in the universe. It is a living and striking truth. The real advance of science will always be in the direction to which established fact points, and he who retraces the line of that advance will encounter every genuine observation which has preceded.

In the last number of this journal we published a full abstract of a paper by Dr. Heitzmann, entitled, Microscopical Studies on Inflammation of the Skin, which seems to mark an epoch in the progress of anatomico-pathological investigation. There are few, if any men in this country, capable of giving as lucid and original an exposition of the subject which he has so carefully studied. Though his paper might, in one light, be considered as chiefly interesting to the members of the Association to whom it was read, it yet in itself demonstrates the fallacy of supposing that any one organ of the body can be studied to the exclusion of others. It opens to the consideration of the profession at large, questions of the widest importance and interest. Much that was expressed by the writer was the result of original investigation. Regarding it, however, for the moment, as the latest exposition of the fruit of the labor of several observers, one can not fail to notice the change it foreshadows in accepted doctrines as to the ultimate constitution of living matter, and the performance of that matter when in a state of inflammation.

For more than a quarter of a century, the medical mind has regarded the "cell" as the single fact of importance in the animal economy, both in health and disease. The term, "cell,"

involves the idea of an enclosed space or cavity. What questionings and searchings has the word not occasioned, since the object it signified first became defined through the primitive objective! Had it an enclosing wall? Had it an enclosed cavity and contents? Did the "cell" produce the "nucleus;" or the "nucleus" the "cell?" In what relation stood "nucleus" and "nucleolus?" Was it reproduced by fission, or by the escape into the surrounding space of its so-called "granules?" These are not a tithe of the problems which rapidly presented themselves.

The result was that the "cell" came to be regarded as the sole unit, potential for good or ill, in every organ or tissue of the body. As Sir Thomas Browne, regarding the macrocosm, of the great world around him, found a still greater when he looked into the microcosm, or little world, of his physical frame, so, in the medical view, the "cell" became the still greater microcosm of the entire body, an autonomous, reproductive and energetic unit from which the macrocosm was built.

Nor with this, were we content. . The "cell," source of all vital activity, became also the active agent in disease. It carried pestilence from man to man, and even from animals to man, and the reverse. "Cancer cells" invaded this tissue; "syphilis cells" that. "Giant cells" threatened here; "small cells," there. We found "broods of cells," "nests of cells," law-abiding and law-breaking "cells."

Exported from the body, they possessed, too, a wonderful activity. They floated in the air and were inhaled of men. They swam in water, and were swallowed by men. In turn, also, they were expelled from all the excretory canals. They were spat, vomited and coughed up. They were cast out from every viscus, whose contents escaped to the external world. Nor was this all. "Cells" of the vegetable world were thought to play their part in the production of disease. There was a plant whose "cells" caused measles; another begat malaria; still another, Asiatic cholera. 'Twas enough to satisfy the soul of Hahnemann himself, who divided all disorders into external and internal itch.

But, a few years ago, certain "cells" were seen in the mucous layer of the epidermis, notably those lying near the papillary layer of the corium, which possessed peculiar projecting spines or points. This fact was held to mean merely, that a new kind of "cell" had been discovered. Yet the significance of the spines or points was not clear. One observer thought they were the media of communication between the "cells" and the nerves; another that the prickles of the so-called "prickle cells" were lymphatic radicles; still another declared that the "prickle cells" hung together by their points; and two microscopists decided that the spines or thorns were interlocked, like the teeth of two adjacent cog-wheels.

Finally, the truth dawned. The spines, prickles, thorns or projecting points were found to be an integral part of the bodies with which they were connected. These bodies were not "cells," but masses of living matter, protoplasmic bodies. They were constituted of a delicate reticulum of living matter, the points of whose intersection formed the so-called "nucleus," "nucleolus" and "granules." Each of these masses of living matter was uninterruptedly united with each adjacent mass of living matter by innumerable spokes or threads of similar constitution. Nor was this true of the rete only. These cords penetrated to the corium, and thus it became evident that the epidermis was joined to the derma in the bonds of a most intimate union. Every mass of living matter was thus in direct connection with every other mass of living matter in the skin. The microcosm was but a part of the macrocosm, after all. The living human body was the real unit. It was found to be clad in a most cunningly devised cloak, a wonderful web of life, with living matter developed most at the points of intersection of warp and woof. In the lifeless cement substance filling the meshes of the web, there were but beaded nerve-fibers of great delicacy, and channels where a nutritive fluid might flow.

In the former belief, the relation of the tissue-elements was illustrated by the isolated cells built in its hive by the honey-bee. The new demonstration represents the living matter by the comb of the same insect, with a lifeless cement-substance in its meshes, represented by the honey.

Who will say that this relationship can be seen only in the skin! It is but reasonable to believe that it extends to all the tissues of animal life.

Dr. Heitzmann gives us the first fruits of these valuable facts, in his discoveries relating to the inflammatory process.

He leads us beyond the "exudation and pus-cells" of Rokitansky; past the "cellular pathology" of Virchow; further even than travel those arrant tramps, the vagrant leucocytes of Cohnheim; leaving far on one side the position of Stricker, who taught that each of his predecessors had a partial glimpse of the truth.

Dr. Heitzmann shows that the process of inflammation is essentially one which concerns the living matter itself. In its earliest stage, the epithelia, or protoplasmic bodies, become swollen, thickened and coarsely granular, in circumscribed spots, as a consequence of the increase of living matter within the bodies themselves, and an augmented influx of nutritive material. The points of intersection of the living reticulum, hitherto known as "granules," become enlarged, as well as the threads which traverse the cement-substance. Dilatation and engorgement of the vascular capillaries in the neighboring papillae ensue, while the protoplasmic material here also undergoes a change similar to that noted in the rete. The connective-tissue bundles are also transformed into protoplasm. In this stage there is no proof of exudation beyond the process of liquefaction of the gluey basis-substance, of which the bundles are composed. The subsequent evolution of this process, as described by the author, is in exactly the course to which the first step here described points, and is in complete accord with clinical experience. But, for fuller details, from which it appears that many of the phenomena hitherto considered essential to the process of inflammation are really epiphenomena, our readers must be referred to the author's text.

If the "cell-doctrine" be threatened with destruction, we may rest assured that from its ruins will rise a clearer appreciation of the interdependence of every part of the organism, both in physiological and pathological states. Every hitherto-established fact will stand. Many unsupported theories will fall. They who have looked on with incredulity and impatience, while men have sought absolution for physical errors in the earth, the air and the water around them, may find that day one of promise.

One might almost believe that the English laureate, in a spirit of poetic prophecy, penned the lines:

"The tiny cell is forlorn,—
Void of its little living will."

